# Thermally Activated Shape-Memory Behavior of SLA-Printed PDMS–PCLDMA Hybrid Networks

**DOI:** 10.3390/polym18172095

**Published:** 2026-08-28

**Authors:** Yura Choi, Namchul Cho

**Affiliations:** Department of Energy Engineering, Soonchunhyang University, 22 Soonchunhyang-ro, Asan 31538, Republic of Korea; bnbn3237@gmail.com

**Keywords:** 4D printing, shape memory polymers, stereolithography, polycaprolactone dimethacrylate, thermally activated shape memory, silicone-acrylate resin

## Abstract

Vat photopolymerization offers a powerful route for high-resolution 4D printing; however, designing shape memory polymers that simultaneously exhibit printability, mechanical robustness, dimensional fidelity, and efficient thermally triggered shape memory behavior remains challenging. In this study, an SLA-printable silicone–semicrystalline hybrid photoresin based on polydimethylsiloxane (PDMS-MMA), polycaprolactone dimethacrylate (PCLDMA), triethylene glycol dimethacrylate (TEGDMA), and trimethylolpropane trimethacrylate (TMPTMA) was developed. The effects of PDMS-MMA and TMPTMA contents on network structure, dimensional stability, mechanical properties, and shape-memory behavior were investigated. Increasing PDMS-MMA content reduced linear shrinkage from 2.1% to 1.7%, suggesting improved dimensional stability associated with increased network flexibility, whereas increasing TMPTMA content enhanced network compactness and reduced swelling. P20T5 exhibited a shape fixity of 95.96% and a recovery ratio of 99.07% in the second cycle. These results demonstrate that balancing flexible silicone segments, semicrystalline switching domains, and covalent network constraints enables the fabrication of SLA-printable hybrid networks with thermally responsive shape-memory behavior.

## 1. Introduction

Additive manufacturing (AM) has emerged as a transformative technology for the fabrication of complex and customized structures that are difficult to achieve through conventional manufacturing methods such as molding or casting [1,2,3]. Among various AM techniques, vat photopolymerization processes including stereolithography (SLA) and digital light processing (DLP) have gained significant attention due to their high printing [4] resolution, excellent surface quality, and compatibility with a wide range of photocurable resins [5,6,7,8]. By integrating stimuli-responsive materials into these systems, the concept of four-dimensional (4D) printing has been introduced, enabling printed structures to dynamically change their shape or functionality in response to external stimuli such as heat, light, humidity, or magnetic fields [9,10,11,12,13,14]. A key class of materials enabling 4D printing is shape memory polymers (SMPs), which can recover a programmed permanent shape after undergoing large temporary deformation [15,16,17]. The shape memory effect typically relies on a dual-network architecture consisting of (i) a permanent network, which stores elastic strain energy and defines the original geometry, and (ii) a switching segment, which temporarily fixes the deformed shape through reversible physical transitions such as glass transition or crystallization [9,18]. In the present hybrid resin system, the covalently crosslinked PDMS-MMA/PCLDMA/TEGDMA/TMPTMA network functions as the permanent network, while the semicrystalline PCLDMA domains act as reversible switching segments. During programming, the PCLDMA crystalline domains are disrupted above their transition temperature, allowing deformation. Upon cooling, recrystallization of PCLDMA temporarily fixes the deformed shape, whereas reheating activates the elastic restoring force stored in the permanent network, leading to recovery of the original geometry. When heated above the transition temperature, the switching domains become mobile, allowing deformation; upon cooling, the domains reform and lock the temporary shape, and reheating subsequently triggers shape recovery [12,17,19,20]. Among various elastomeric materials, polydimethylsiloxane (PDMS) is particularly attractive as a permanent network component due to its exceptional flexibility, chemical stability, low glass transition temperature, and biocompatibility [21,22,23]. These characteristics make PDMS-based systems promising candidates for soft robotics, biomedical devices, and adaptive structures [22,24]. However, the high chain mobility and low modulus of PDMS networks often lead to insufficient shape fixation, as the soft silicone chains cannot effectively resist the entropic restoring force generated during deformation [4,21]. Consequently, designing silicone-based SMPs requires carefully balancing elastic energy storage and temporary shape fixation, which remains a significant challenge in current material systems [22,24,25,26].

To overcome these limitations, semicrystalline switching segments have been incorporated into elastomeric networks to provide reversible physical crosslinks. In particular, polycaprolactone dimethacrylate (PCLDMA) is an attractive switching segment for photocurable shape memory systems because it combines the crystallizable nature of polycaprolactone with acrylate end groups that enable photo-crosslinking [27,28,29,30]. In crosslinked networks, the PCL segments form reversible crystalline domains that act as temporary physical crosslinks, allowing deformation above the melting transition and fixation of temporary shapes upon cooling through recrystallization [31,32]. Owing to its semicrystalline character, biocompatibility, biodegradability, and compatibility with vat photopolymerization, PCLDMA has been widely used in SLA/DLP-type photocurable resin systems [28,30,31]. Previous studies have shown that PCLDMA-based formulations can be successfully processed by light-based 3D printing and can exhibit effective shape-fixity and recovery behavior, supporting its suitability as a thermally responsive switching segment in 4D printing applications [20,24,27,33]. Despite these advantages, incorporating semicrystalline switching segments into photocurable elastomeric networks introduces an inherent competition between covalent crosslinking and crystallization [25,34]. Increasing crosslink density improves elastic energy storage and shape recovery efficiency, but simultaneously restricts the chain mobility required for PCL crystallization, thereby reducing shape fixation capability [32,35]. Conversely, excessive crystallinity or high oligomer content may impair resin homogeneity, viscosity, and processability during vat photopolymerization. Therefore, achieving an appropriate balance between network elasticity, crystallization behavior, and printability is essential for designing high-performance SMP systems suitable for high-resolution 4D printing [36,37,38,39].

**Figure 1:**
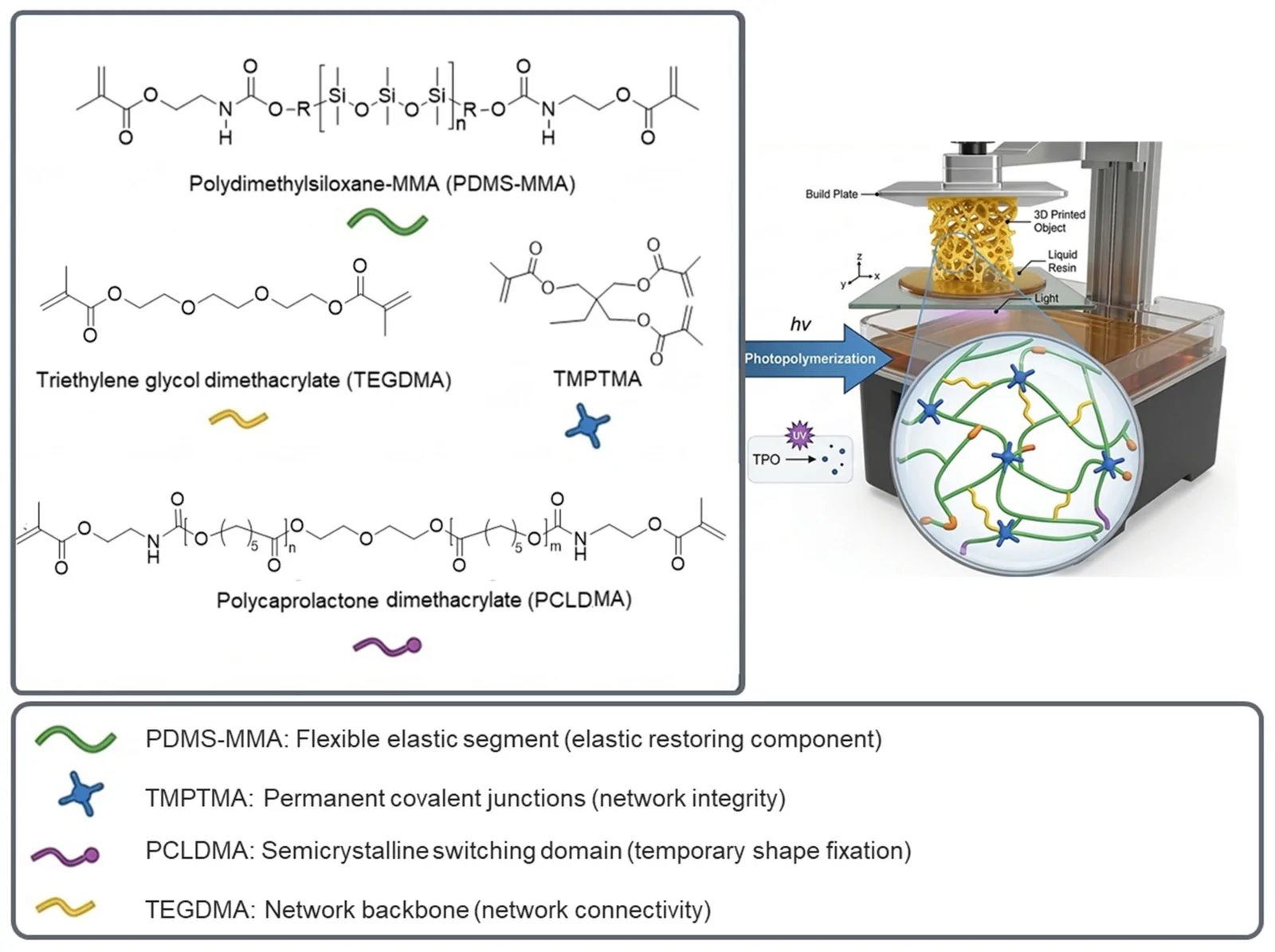
Network design of the SLA-printable silicone–PCLDMA hybrid resin. The chemical structures of the components were prepared using ChemDraw Ultra 12.0.

In this work, we investigate an SLA-printable hybrid photoresin based on PDMS-MMA, PCLDMA, TEGDMA, TMPTMA, and a photocurable urethane acrylate oligomer. The formulation matrix was designed to examine composition-dependent relationships among network formation, dimensional fidelity, thermomechanical behavior, mechanical properties, and thermally activated shape-memory response. In contrast to treating PDMS-MMA and TMPTMA as strictly independent design variables, the present study considers the resulting properties at the formulation level because changes in these components were accompanied by compensating changes in TEGDMA content. Particular attention was given to integrating a silicone-containing component with a PCLDMA-based photocurable network while maintaining compatibility with SLA-based vat photopolymerization. The study therefore aims to establish practical formulation–processing–property relationships for thermally responsive hybrid networks rather than to assign the observed behavior to a single independently controlled network component. Compared with previous PCL-derived vat-photopolymerized shape-memory systems, the present study specifically examines the combined influence of a methacrylate-functionalized silicone component and crosslinker content on printability, network characteristics, dimensional stability, and shape-memory performance. The overall resin design and network concept are illustrated in Figure 1.

## 2. Materials and Methods

### 2.1. Materials

PDMS-MMA (methacrylate-functionalized polydimethylsiloxane) was used as the soft elastic domain to provide energy storage capability. Polycaprolactone dimethacrylate (PCLDMA), a crystallizable PCL-derived photocurable component, and triethylene glycol di-methacrylate (TEGDMA), the diacrylate backbone for network regulation, were employed as primary components. Trimethylolpropane trimethacrylate (TMPTMA) was used as a trifunctional crosslinker to establish permanent network points. Diphenyl(2,4,6-trimethylbenzoyl)phosphine oxide (TPO) was utilized as the photoinitiator for 405 nm UV curing. PDMS-MMA (M_n_ = 1300–1400 g/mol) and PCLDMA (M_n_ = 2400–4000 g/mol) were purchased from Burkgilman Co., Ltd. (Asan, Republic of Korea). TMPTMA and TPO were obtained from Miwon Chemical Co., Ltd. (Gyeonggido, Republic of Korea). TEGDMA was purchased from Bld pharm Co., Ltd. (Shanghai, China). The bi-functional aliphatic urethane acrylate oligomer was obtained from Miwon Chemical Co., Ltd. (Yongin, Republic of Korea). All chemicals were used as received without further purification.

### 2.2. Resin Formulation

Photoresin formulations were designed to investigate the interplay between PCLDMA-based semicrystalline switching and network crosslinking density. Two formulation groups were prepared. One formulation series varied the PDMS-MMA content (0, 10, and 20 wt%) while maintaining a constant TMPTMA content of 5 wt%. The second series varied the TMPTMA content (5, 10, and 15 wt%) at a fixed PDMS-MMA content of 10 wt%. The components were weighed according to the compositions listed in Table 1 and mixed in amber glass vials to prevent premature photopolymerization. The mixture was dispersed via ball milling at 200 rpm for 12 h at 25 ± 1 °C under light-shielded conditions to prevent unintended photopolymerization. The final resins appeared as transparent liquids suitable for vat photopolymerization. All compositions in Table 1 are expressed as weight percentages of the total resin formulation, including 2 wt% TPO, and each formulation was normalized to 100 wt%.

The viscosity of each resin was measured using a rotational viscometer (DV2TLV Rheometer, Brookfield, Middleboro, MA, USA) at a controlled temperature of 24 ± 1 °C. Viscosity measurements were performed at a constant rotation speed of 60 rpm for 2 min, and the results were recorded in centipoise (cP). Each formulation was assessed in triplicate, and the mean value was reported.

### 2.3. 3D Printing and Post-Processing Conditions

Three-dimensional printing was performed using an A+ SLA 3D printer (Shindoh Co., Ltd., Seoul, Republic of Korea) equipped with a 405 nm laser diode (nominal output power: 600 mW). The layer thickness was set to 50 μm, and all samples were printed under identical processing parameters to enable direct comparison between resin formulations. The printing conditions were selected based on preliminary printing trials to achieve stable fabrication of test specimens. Since this study focused on the influence of resin formulation rather than printing parameter optimization, identical printing parameters were applied to minimize processing-related variations. A systematic optimization of printing parameters was beyond the scope of this work. The printed specimens were oriented horizontally on the build platform to minimize structural distortion during fabrication.

After printing, the specimens were rinsed with ethanol to remove uncured resin and subsequently post-cured under a 405 nm UV curing system (80 W) for approximately 5 min at room temperature to promote further curing and network stabilization.

### 2.4. Characterization of Network and Thermal Properties

#### 2.4.1. FT-IR, Gel Fraction, and Swelling Measurements

FT-IR spectroscopy was used to qualitatively assess changes in the methacrylate-related absorption bands before and after UV curing (FT-IR, Nicolet iS50, Thermo Scientific Madison, WI, USA). Spectra were recorded in the range of 4000–600 cm^−1^ with a resolution of 4 cm^−1^. Changes in the methacrylate-related C=C absorption band near 1638 cm^−1^ were qualitatively examined before and after UV curing, while the C=O band near 1715 cm^−1^ and the Si–O–Si band near 1080 cm^−1^ were used to identify characteristic functionalities of the acrylate/methacrylate and siloxane-containing components.

Gel fraction was determined by Soxhlet extraction using tetrahydrofuran (THF) as the solvent for 24 h to remove uncrosslinked polymer fractions. The extracted samples were then dried under vacuum at 60 °C until constant weight. Unless otherwise stated, quantitative data are presented as mean ± standard deviation (*n* = 3)

(1) $$Gel\;fraction\;(\%)=m_{d}{/m}_{i}\times 100$$

*m_i_*: Initial dry mass before extraction. *m_d_*: Dried mass after extraction.

Swelling ratio measurements were conducted by immersing dried samples in THF at room temperature for 24 h until equilibrium swelling was reached. Unless otherwise stated, quantitative data are presented as mean ± standard deviation (*n* = 3). The swelling ratio was calculated using:

(2) $$Swelling\;ratio\;=\;{(w}_{s}-w_{d}){/w}_{d}$$

*w_s_*: Swollen mass after equilibrium. *w_d_*: Dried mass after swelling measurement.

Linear shrinkage was evaluated using square specimens (20 mm × 20 mm) fabricated by SLA 3D printing under identical processing conditions. The designed side length (L_0_ = 20 mm) was compared with the final dimension (L) measured after print-ing and UV post-curing. Dimensional measurements were carried out using a digital caliper with an accuracy of ±0.01 mm. Unless otherwise stated, quantitative data are presented as mean ± standard deviation (*n* = 3). Linear shrinkage (%) was calculated using the following equation:

(3) $$Linear\;shrinkage\;\left(\%\right)\;=\;({(L}_{0}-L)/L_{0})\times 100$$

#### 2.4.2. Thermogravimetric Analysis (TGA)

The thermal stability of the cured PDMS–PCLDMA hybrid networks was evaluated using thermogravimetric analysis (TGA). TGA was performed using a thermogravimetric analyzer (Q50, TA Instruments, New Castle, DE, USA), in which approximately 10–20 mg of each cured sample was heated from room temperature to 500 °C at a heating rate of 10 °C min^−1^ under a nitrogen atmosphere.

#### 2.4.3. Dynamic Mechanical Analysis (DMA)

Dynamic mechanical properties were evaluated using a dynamic mechanical analyzer (DMA Q800, TA Instruments) in tensile mode. Rectangular specimens (dimensions: 20 mm × 5 mm × 1 mm) were tested at a frequency of 1 Hz with a temperature ramp from 25 °C to 140 °C at a heating rate of 5 °C/min. The storage modulus (E′), loss modulus (E″), and loss factor (tan δ) were used to evaluate the thermomechanical behavior and temperature dependent network mobility of the printed networks.

### 2.5. Mechanical and Shape-Memory Characterization

#### 2.5.1. Tensile Testing

Uniaxial tensile tests were conducted using a universal testing machine (AGS-X, Shimadzu, Kyoto, Japan) equipped with a 10 kN load cell in accordance with ASTM D638 standards [40]. Dogbone shaped specimens were printed with dimensions of 63 mm × 5 mm × 2 mm and a gauge length of 10 mm. Tests were performed at a crosshead speed of 10 mm/min at room temperature (*n* = 3).

#### 2.5.2. Shape Memory Test (SMT)

Shape memory behavior was evaluated using a cyclic thermomechanical testing protocol under force-control mode. Force-control mode was selected because stable strain-controlled programming was not achievable for the printed specimens during preliminary testing. The specimens were first heated to 90–100 °C, which is above the switching transition of the PCLDMA-containing network, and equilibrated before mechanical programming. A predefined force was then gradually applied to deform the specimen. After reaching the target force or target deformation, the applied force was maintained while the specimen was cooled to approximately 20 °C to fix the temporary shape. Here, ε_load_, ε_fix_, and ε_rec_ represent the strain after loading, after fixing, and after recovery, respectively. The quantitative shape-memory parameters were evaluated using rectangular specimens, while the recovery behavior of the 3D printed lattice structures was qualitatively demonstrated separately to show the applicability of the developed resin.

(4) $$R_{f}(N)=\varepsilon_{fix}(N)/\varepsilon_{load}\left(N\right)\times 100$$

(5) $$R_{r}(N)=\frac{\left[\varepsilon_{load}\left(N\right)-\varepsilon_{rec}\left(N\right)\right]}{\left[\varepsilon_{load}\left(N\right)-\varepsilon_{rec}\left(N-1\right)\right]}\times 100$$

*R_f_*: Shape fixity ratio. *R_r_*: Shape recovery ratio.

## 3. Results and Discussion

### 3.1. Resin Viscosity and Qualitative Assessment of Photopolymerization

**Figure 2:**
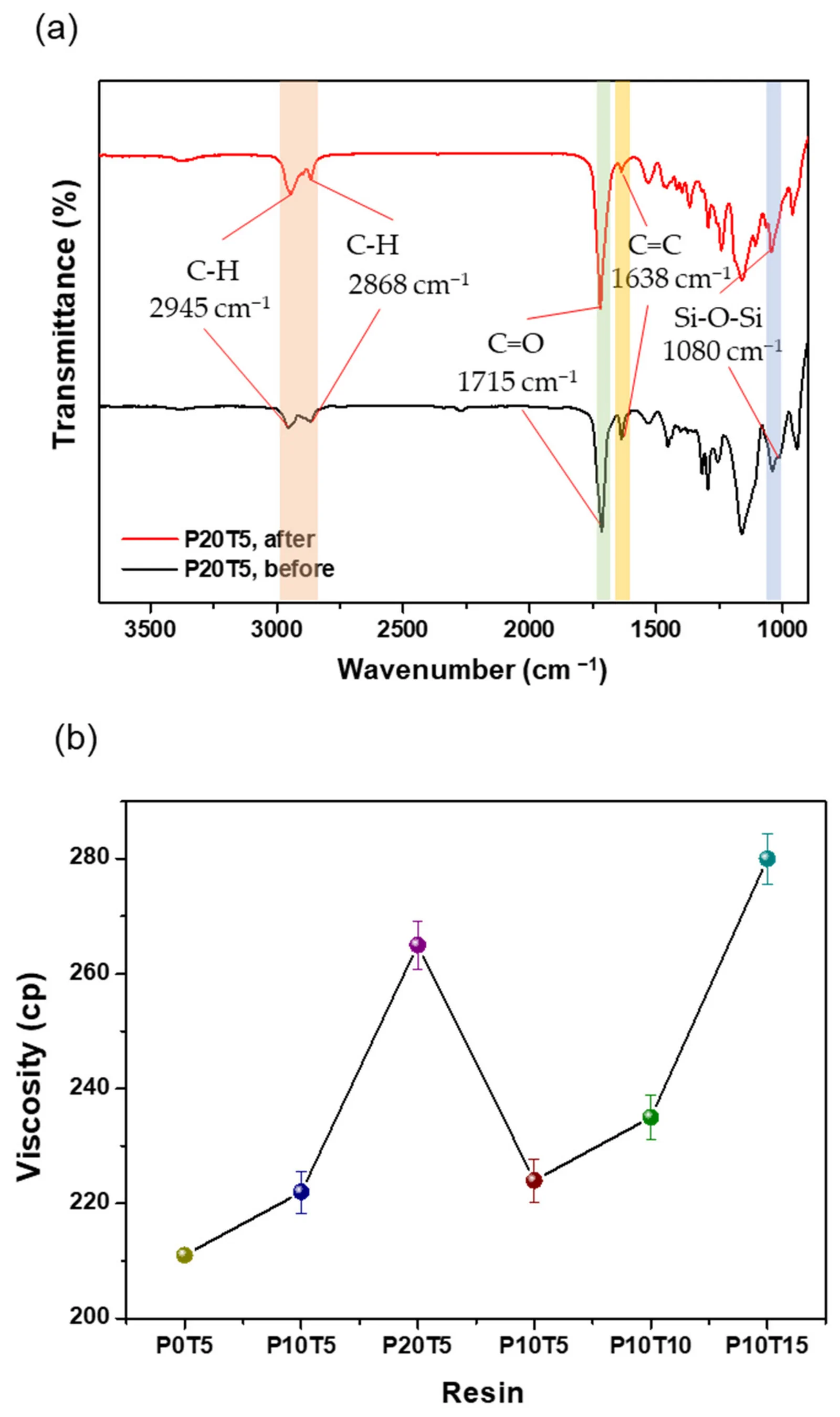
(**a**) FT-IR spectra of the P20T5 formulation before and after UV curing, showing the decrease in the acrylate C=C absorption band at approximately 1638 cm^−1^ and the presence of characteristic C=O and Si–O–Si bands. (**b**) Viscosity of the PDMS–PCLDMA hybrid resin formulations with different PDMS-MMA and TMPTMA contents.

The rheological behavior and photopolymerization efficiency of the formulated PDMS–PCLDMA hybrid photoresins were first evaluated to confirm their suitability for stereolithography (SLA) processing. As shown in Figure 2, the resin formulations exhibited viscosities within a printable range for vat photopolymerization. The viscosity varied depending on the relative contents of PDMS-MMA and TMPTMA, reflecting the combined effects of the flexible siloxane segments, semicrystalline PCLDMA component, and multifunctional methacrylate crosslinker. The incorporation of PDMS-MMA influenced the resin viscosity because of the flexible siloxane backbone and its relatively high molecular weight compared with low molecular weight acrylate monomers. Nevertheless, all formulations remained sufficiently fluid for layer-by-layer recoating during SLA printing. This result indicates that the designed resin system, composed of PDMS-MMA, PCLDMA, TEGDMA, and TMPTMA, maintains adequate processability despite the incorporation of both elastomeric and semicrystalline components. Photopolymerization behavior was analyzed by FT-IR spectroscopy before and after UV curing. The decrease in the C=C-associated absorption near 1638 cm^−1^ qualitatively indicates consumption of photoreactive double bonds during curing. Because quantitative double-bond conversion was not determined, the FT-IR results are interpreted as qualitative evidence of photopolymerization. In addition, the carbonyl stretching band near 1715 cm^−1^ and the Si–O–Si absorption associated with PDMS-MMA near 1080 cm^−1^ were observed, supporting the incorporation of both acrylate/methacrylate and siloxane components in the cured hybrid network. The FT-IR results therefore provide qualitative evidence of photopolymerization under 405 nm irradiation. The formation of a covalently crosslinked network is attributed to the reaction of methacrylate/acrylate groups in PDMS-MMA, PCLDMA, TEGDMA, and TMPTMA. In this network, PDMS-MMA serves as the flexible elastic domain, PCLDMA provides semicrystalline switching segments, TEGDMA contributes to the structural backbone, and TMPTMA introduces multifunctional crosslinking points. Together, the qualitative FT-IR observations and measured viscosity support the processability of the investigated resin formulations by vat photopolymerization. Overall, the viscosity and qualitative FT-IR results support the processability and network-forming capability of the investigated resin system. This processing feasibility provides the basis for subsequent evaluation of network characteristics, thermomechanical properties, and one-way shape-memory behavior. 

### 3.2. Formation, Swelling Behavior, and Dimensional Fidelity

**Figure 3:**
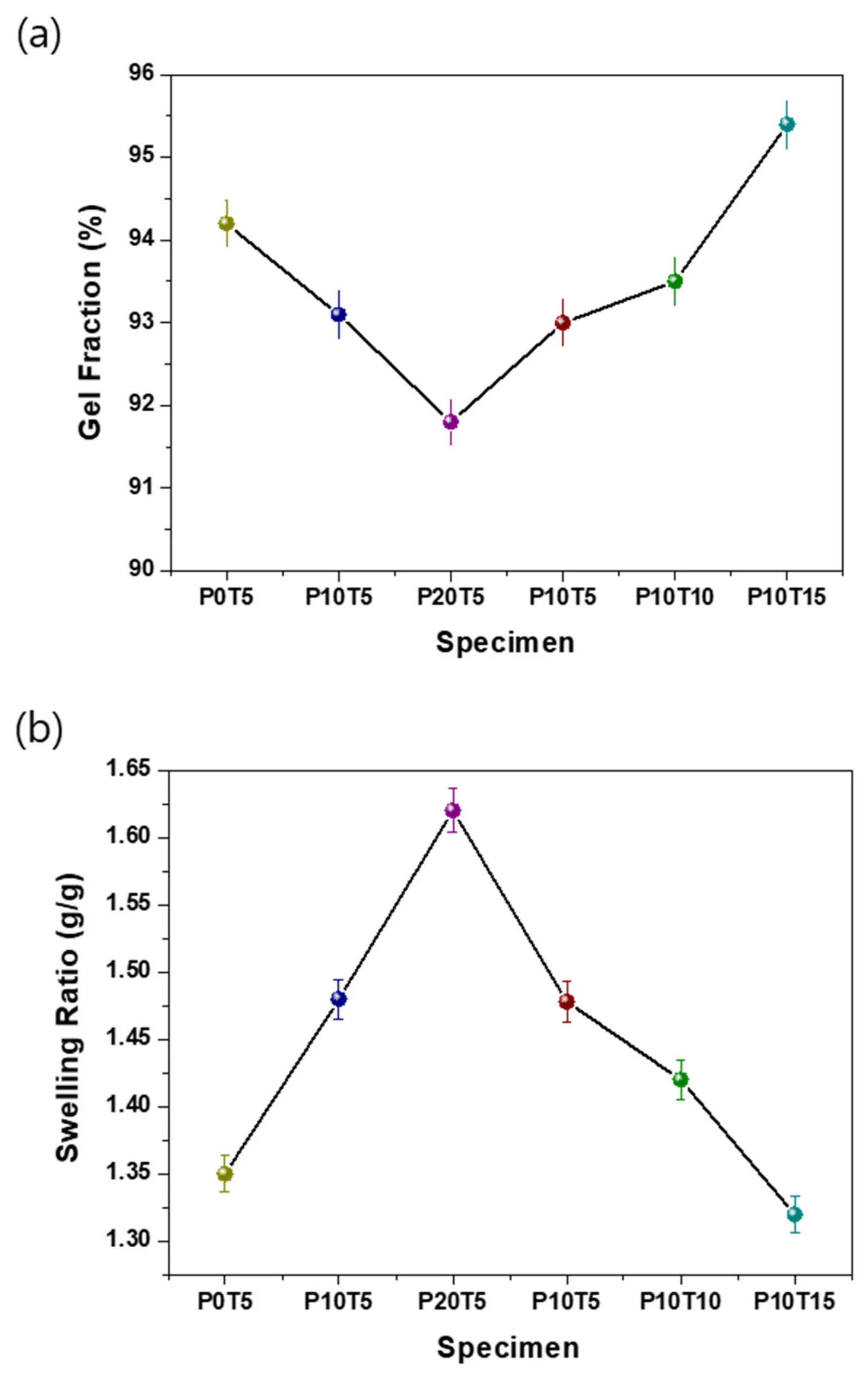
(**a**) Gel fraction of the SLA-printed PDMS–PCLDMA hybrid networks after THF extraction, showing the effect of PDMS-MMA content. (**b**) Equilibrium swelling ratio of the same formulations in THF, reflecting changes in network compactness and solvent uptake with increasing PDMS-MMA or TMPTMA content.

The network structure of the SLA-printed PDMS–PCLDMA hybrid materials was evaluated by gel fraction and equilibrium swelling measurements. These parameters provide complementary information on the degree of covalent network formation and the relative compactness of the photocured polymer networks. A high gel fraction indicates effective incorporation of photocurable components into the insoluble crosslinked network, whereas the swelling ratio reflects the ability of solvent molecules to penetrate the network and is inversely related to the effective crosslink density. As summarized in Table 2 and Figure 3, all printed samples exhibited high gel fractions above 90%, confirming successful network formation after SLA printing and post-curing. This result indicates that the methacrylate/acrylate groups of PDMS-MMA, PCLDMA, TEGDMA, and TMPTMA were efficiently polymerized to form stable covalent networks. Although the formulations contained both flexible PDMS-MMA segments and semicrystalline PCLDMA domains, no severe loss of network integrity was observed, demonstrating that the resin composition was suitable for photocuring-based fabrication.

#### 3.2.1. Effect of PDMS-MMA Content on Network Structure

Where the PDMS-MMA content was varied from 0 to 20 wt% at a fixed TMPTMA content of 5 wt%, the gel fraction gradually decreased from 94.2 ± 0.5% for P0T5 to 93.1 ± 0.8% for P10T5 and 91.8 ± 1.1% for P20T5. In contrast, the swelling ratio increased from 1.35 ± 0.04 g/g for P0T5 to 1.48 ± 0.06 g/g for P10T5 and 1.62 ± 0.09 g/g for P20T5. This trend suggests that increasing the PDMS-MMA content slightly reduces the effective network compactness. The decrease in gel fraction with increasing PDMS-MMA content can be attributed to the relatively flexible siloxane backbone and the dilution of more rigid multifunctional methacrylate components in the formulation. PDMS-MMA introduces highly mobile chain segments into the network, which can increase free volume and facilitate solvent uptake. Consequently, the higher swelling ratio observed for P20T5 indicates a more flexible and loosely packed network compared with P0T5. Nevertheless, the gel fraction remained above 90% even at 20 wt% PDMS-MMA, confirming that PDMS-MMA was successfully incorporated into the photocured network without causing significant sol fraction formation. This behavior is important for shape memory performance because PDMS-MMA contributes to elastic recovery by providing flexible network segments. Furthermore, the increased swelling capability may influence shape-memory performance by providing enhanced segmental mobility during thermal deformation and recovery. The higher swelling ratio of P20T5 suggests reduced network constraint, which may facilitate segmental mobility during thermally activated recovery. However, excessive PDMS-MMA may reduce network compactness and weaken the ability of the material to maintain a fixed temporary shape. Therefore, the PDMS-MMA content must be balanced to provide sufficient elastic mobility without compromising network stability.

#### 3.2.2. Effect of TMPTMA Content on Network Compactness

The effect of crosslinker concentration was examined at a fixed PDMS-MMA content of 10 wt%. As the TMPTMA content increased from 5 to 15 wt%, the gel fraction increased from 93.1 ± 0.8% for P10T5 to 93.5 ± 0.8% for P10T10 and 95.4 ± 0.4% for P10T15. At the same time, the swelling ratio decreased from 1.47 ± 0.06 g/g to 1.42 ± 0.04 g/g and 1.32 ± 0.05 g/g, respectively. This inverse relationship between gel fraction and swelling ratio clearly indicates that TMPTMA effectively increases the crosslink density of the hybrid network. Because TMPTMA is a trifunctional methacrylate crosslinker, increasing its concentration provides more covalent junction points during photopolymerization. As a result, the network becomes more compact and less accessible to solvent penetration. The reduced swelling ratio of P10T15 therefore reflects the formation of a denser network structure compared with P10T5 and P10T10. However, a higher crosslink density is not necessarily beneficial for all functional properties. In semicrystalline shape memory networks, excessive covalent constraint can restrict the mobility of PCLDMA segments and hinder their crystallization during cooling. Since PCLDMA crystalline domains are responsible for temporary shape fixation, over-crosslinking may reduce shape fixity even though it improves network integrity. Thus, the TMPTMA content must be optimized to provide enough permanent network points for shape recovery while preserving sufficient PCLDMA chain mobility for crystallization driven shape fixing.

#### 3.2.3. Dimensional Fidelity and Linear Shrinkage

**Figure 4:**
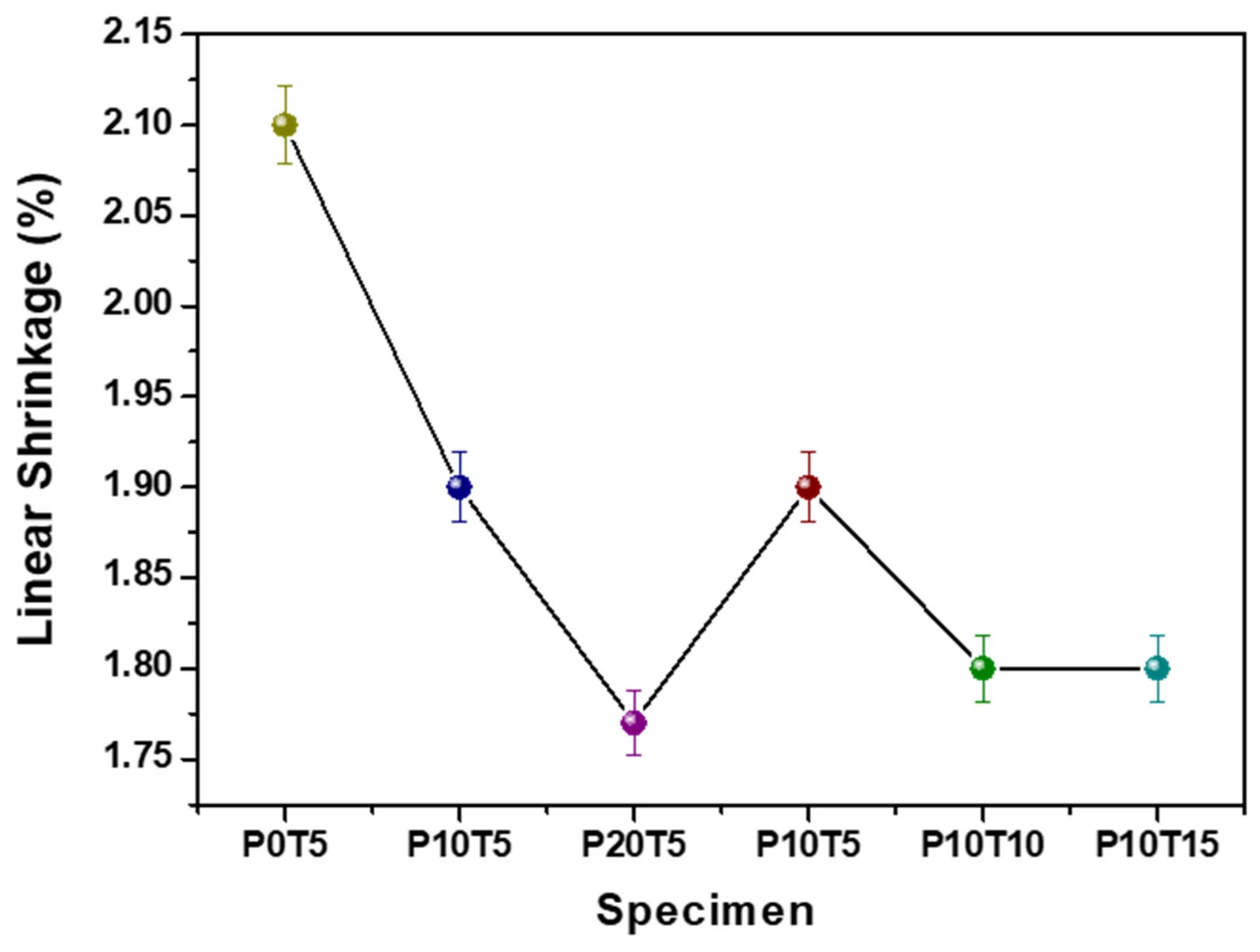
Linear shrinkage of SLA-printed PDMS–PCLDMA hybrid networks, showing the effects of PDMS-MMA and TMPTMA contents.

Dimensional fidelity is a critical requirement for SLA-printed shape memory materials because polymerization shrinkage can distort the designed geometry and reduce the accuracy of programmed shape transformation. The linear shrinkage results are summarized in Table 3 and plotted in Figure 4. Increasing the PDMS-MMA content reduced the linear shrinkage from 2.1 ± 0.01% for P0T5 to 1.9 ± 0.02% for P10T5 and 1.7 ± 0.01% for P20T5. This reduction can be explained by the flexible and relatively high-molecular-weight siloxane segments of PDMS-MMA, which lower the volumetric contraction associated with the conversion of monomeric double bonds into covalent polymer networks. The incorporation of PDMS-MMA therefore helps relieve internal stress generated during photopolymerization and improves dimensional stability. The linear shrinkage values remained relatively low and similar, with 1.9 ± 0.02% for P10T5, 1.8 ± 0.01% for P10T10, and 1.8 ± 0.02% for P10T15. This indicates that increasing TMPTMA content within the investigated range only slightly affected macroscopic shrinkage. Although TMPTMA increases crosslink density, the overall formulation still maintained low shrinkage, likely because the network contains a significant fraction of high-molecular-weight and flexible components such as PDMS-MMA and PCLDMA. Overall, the network and dimensional analyses demonstrate that the PDMS–PCLDMA hybrid formulations form highly crosslinked yet dimensionally stable SLA-printed networks. PDMS-MMA increases network flexibility and reduces polymerization shrinkage, whereas TMPTMA enhances gel fraction and suppresses swelling by increasing crosslink density. These opposing but complementary effects establish the structural basis for controlling the balance between dimensional fidelity, elastic recovery, and PCLDMA-based shape fixation in the subsequent shape memory behavior. Although comprehensive printability metrics such as surface roughness, dimensional accuracy, and internal porosity were not quantitatively evaluated in this study, the observed shrinkage behavior and successful fabrication of complex lattice structures indicate the feasibility of the developed resin for SLA processing.

### 3.3. Thermal Stability of the PDMS–PCLDMA Hybrid Networks

**Figure 5:**
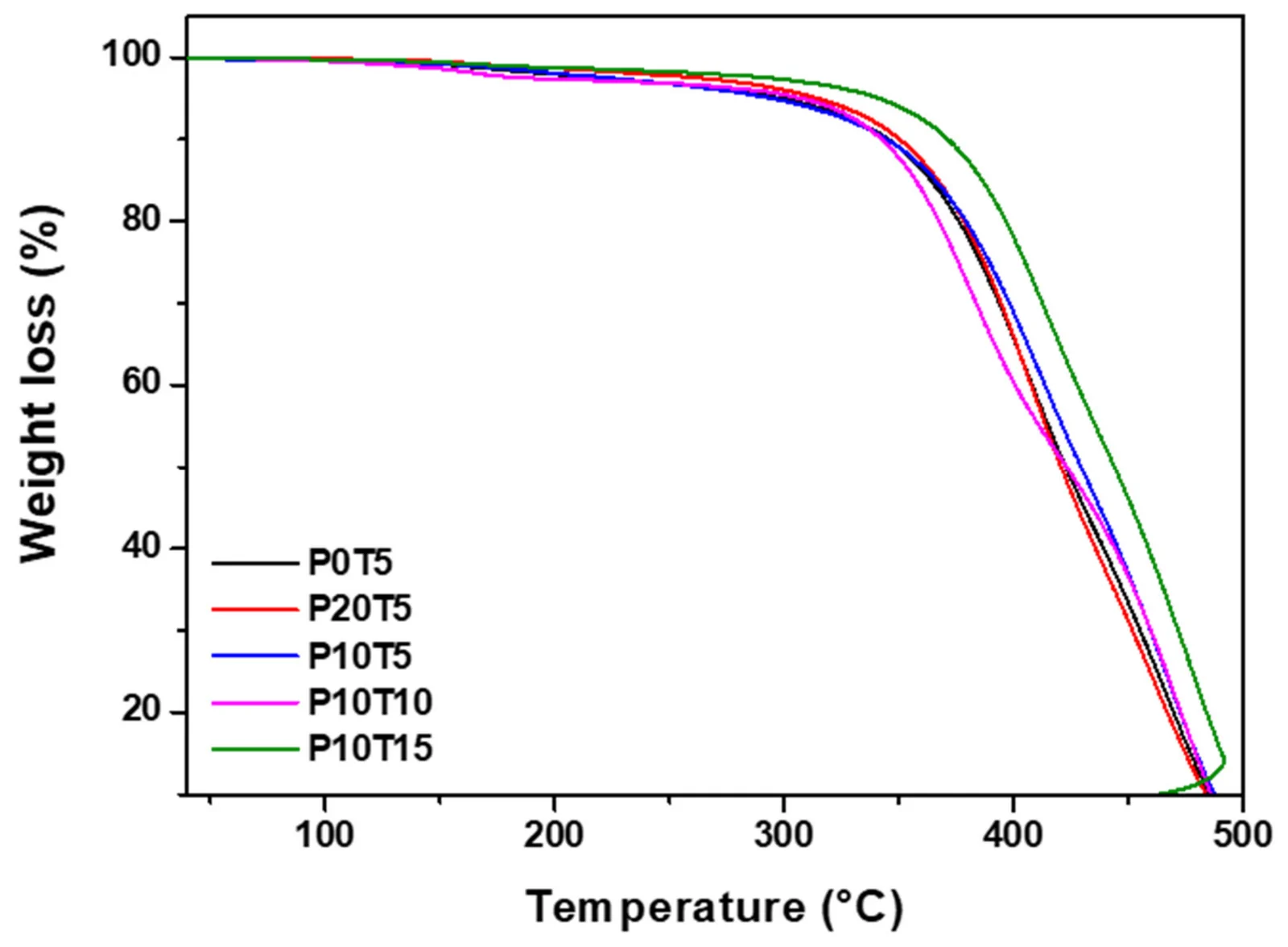
TGA thermograms of the SLA-printed PDMS–PCLDMA hybrid networks.

The thermal stability of the SLA-printed PDMS–PCLDMA hybrid networks was evaluated using thermogravimetric analysis (TGA). Since the proposed materials are designed for thermally triggered shape memory behavior, it is essential to confirm that the polymer networks remain stable within the temperature range used for programming and recovery. In this study, the shape memory process was conducted at temperatures much lower than the thermal degradation region; therefore, TGA was used to verify whether the printed networks possess a sufficient thermal stability window for repeated thermal actuation. As shown in Figure 5, all formulations exhibited stable thermal behavior over the temperature range relevant to shape memory programming and recovery. The major weight-loss region appeared only at elevated temperatures, indicating that the PDMS–PCLDMA hybrid networks did not undergo significant thermal decomposition under the actuation conditions. This result confirms that the observed shape memory response can be attributed to thermally induced molecular mobility and reversible switching of the PCLDMA-containing network rather than irreversible thermal degradation. Where the PDMS-MMA content was varied at a fixed TMPTMA concentration, the TGA curves showed no severe deterioration in thermal stability with increasing PDMS-MMA content. The incorporation of PDMS-MMA may contribute to the thermal resistance of the hybrid network because the siloxane backbone possesses relatively high bond stability. At the same time, the degradation behavior of the printed materials is governed by the combined decomposition of the organic PCLDMA, TEGDMA, and TMPTMA network components. Therefore, the observed TGA response reflects the hybrid nature of the network, consisting of flexible PDMS-MMA domains and photocrosslinked acrylate/methacrylate segments. Increasing the TMPTMA content did not affect the thermal degradation behavior. As discussed in Section 3.2, higher TMPTMA concentration increased the gel fraction and reduced the swelling ratio, indicating the formation of a more compact covalent network. Such a denser network can restrict chain mobility and may contribute to improved thermal resistance. However, within the investigated TMPTMA range, the overall degradation profiles remained similar, suggesting that crosslinker con-centration mainly influenced network compactness rather than drastically changing the degradation pathway. Overall, the TGA results demonstrate that the SLA-printed PDMS–PCLDMA hybrid networks possess sufficient thermal stability for thermally activated shape memory operation. The degradation region is well above the programming and recovery temperatures, confirming that the one-way shape memory behavior occurs within a thermally stable operating window. Therefore, the following thermomechanical and shape memory analyses can be interpreted in terms of network mobility, elastic recovery, and PCLDMA-based switching behavior without concerns regarding thermal degradation during actuation.

### 3.4. Thermomechanical Properties of the PDMS–PCLDMA Hybrid Networks

**Figure 6:**
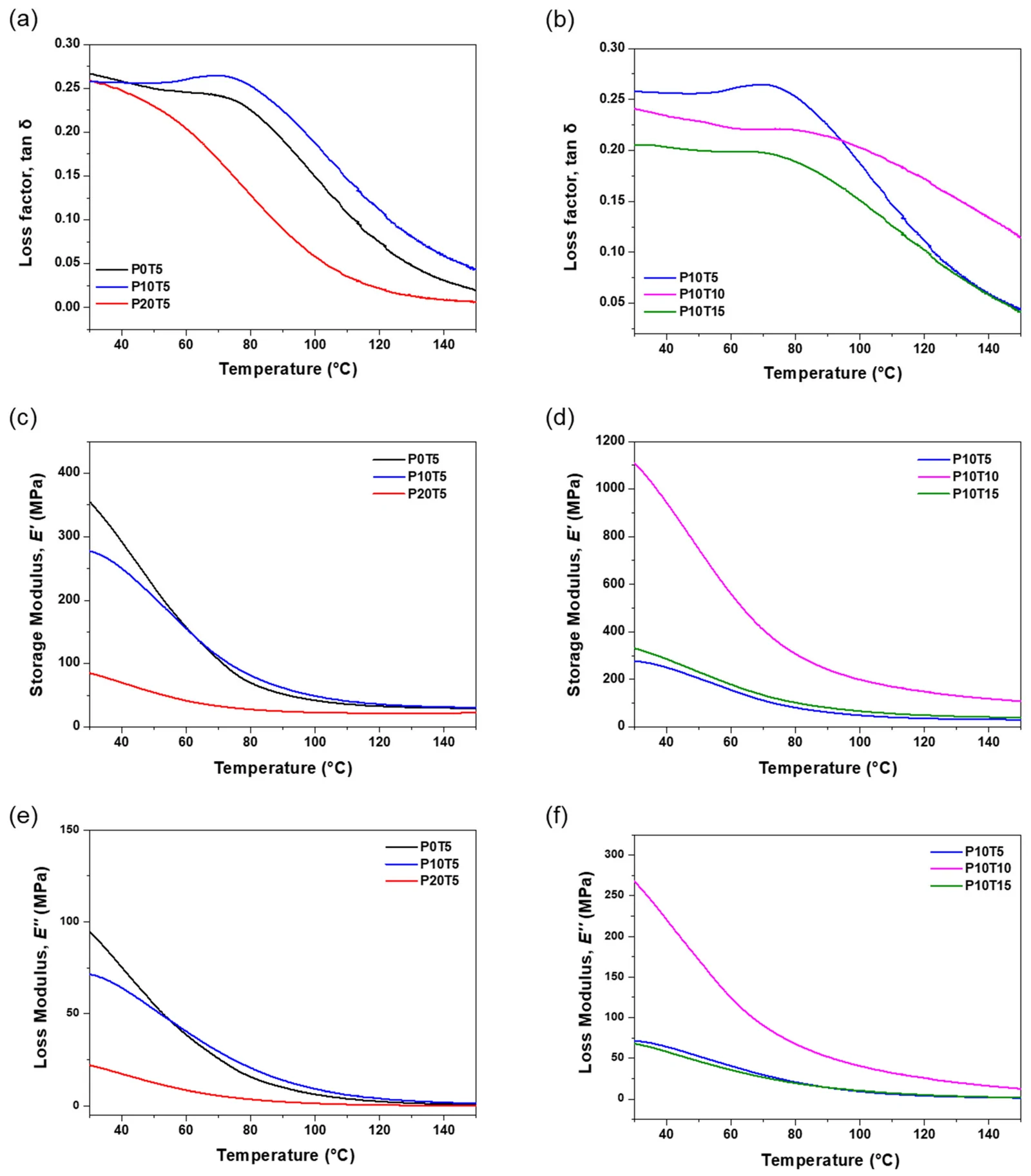
DMA Results. (**a**,**b**) Loss factor, tanδ, (**c**,**d**) Storage modulus E′, (**e**,**f**) Loss modulus E′′. (**a**,**c**,**e**) PDMS-MMA series, (**b**,**d**,**f**) TMPTMA series.

Dynamic mechanical analysis (DMA) was performed to evaluate the thermomechanical response of the SLA-printed PDMS–PCLDMA hybrid networks. The temperature dependent storage modulus (E′), loss modulus (E″), and loss factor (tan δ) provide information on network stiffness, energy dissipation, and thermally induced molecular mobility. In shape memory polymers, these parameters are closely related to the ability of the material to store elastic strain energy during programming and release it during recovery. As shown in Figure 6, the printed hybrid networks exhibited clear temperature dependent viscoelastic behavior. The storage modulus decreased with increasing temperature, indicating thermal softening of the crosslinked network. This modulus reduction is important for shape memory programming because the material must become sufficiently deformable above the switching transition. Upon cooling, the PCLDMA crystalline domains are expected to reform and stabilize the temporary shape, while the covalent PDMS-MMA/TEGDMA/TMPTMA network preserves the permanent shape. The gradual decrease in storage modulus with increasing temperature indicates progressive thermal softening of the photocrosslinked network, which facilitates thermally assisted deformation and subsequent shape recovery. Because PCL-containing networks are known to exhibit semicrystalline thermal transitions, melting and recrystallization of PCLDMA segments may contribute to the observed thermally activated response.

#### 3.4.1. Effect of PDMS-MMA Content on Network Mobility

The PDMS-MMA content was varied at a fixed TMPTMA concentration to examine how the flexible siloxane component affects the thermomechanical behavior of the hybrid networks. PDMS-MMA introduces highly mobile siloxane chains into the photocured network, which can reduce rigidity and improve segmental mobility. This effect is consistent with the swelling results in Section 3.2, where increasing PDMS-MMA content led to higher swelling ratios, indicating a more flexible and less compact network structure. The storage modulus profiles suggest that PDMS-MMA functions as a soft elastic domain within the PDMS–PCLDMA network. Increasing the PDMS-MMA content is expected to lower the overall stiffness or promote a more compliant thermomechanical response, particularly at elevated temperatures where the photocrosslinked network exhibits increased segmental mobility. This enhanced mobility can facilitate shape recovery by allowing the network chains to rearrange more readily during heating. However, the role of PDMS-MMA must be balanced. While flexible siloxane segments can accelerate recovery and reduce internal stress, excessive PDMS-MMA content may decrease the effective network constraint needed to store elastic energy and maintain dimensional stability. Excessive incorporation of flexible segments may alter the balance between network constraint and segmental mobility required for stable temporary-shape fixation. Therefore, PDMS-MMA improves thermomechanical mobility, but its content must be optimized to avoid excessive softening of the network. These observations also provide a possible explanation for the relationship between swelling behavior and shape recovery. This relationship suggests that the swelling behavior can serve as an indirect indicator of network mobility, which may influence the thermally activated recovery process.

#### 3.4.2. Effect of TMPTMA Content on Network Constraint

The TMPTMA concentration was varied at a fixed PDMS-MMA content to evaluate the effect of crosslink density on the thermomechanical response. TMPTMA is a trifunctional methacrylate crosslinker that increases the number of covalent junction points in the photocured network. As confirmed by the gel fraction and swelling measurements, increasing TMPTMA content produced a more compact network structure with higher gel fraction and lower swelling ratio. Increasing TMPTMA incorporation is generally expected to enhance network rigidity by introducing additional multifunctional crosslinking points. However, the storage modulus in the present TMPTMA series did not show a simple monotonic dependence on TMPTMA concentration. This behavior is attributed to the simultaneous variation in TEGDMA content during formulation adjustment, which alters the balance between network crosslinking density and chain mobility. Therefore, the dynamic mechanical response is governed by the overall network architecture rather than TMPTMA concentration alone. Accordingly, the present formulation study was designed to identify composition-dependent trends and structure–property relationships, rather than to develop a statistical prediction model using DOE or RSM. The significantly higher storage modulus observed for P10T10 suggests that this formulation exhibited a favorable balance between network constraint and chain mobility.

The DMA results further support the role of TMPTMA as a network-stiffening component. This increased elastic contribution is beneficial for shape recovery because the permanent network must store sufficient strain energy during programming and drive the recovery process upon reheating. Nevertheless, excessive crosslinking can restrict the mobility of PCLDMA-containing segments. The thermally activated response of the cured hybrid network is therefore more appropriately interpreted in terms of overall segmental mobility and network constraint. An excessively dense covalent network may further restrict segmental mobility during programming, cooling, and recovery, potentially limiting temporary-shape fixation and subsequent recovery. Thus, increasing TMPTMA strengthens the permanent network, whereas excessive network constraint may limit the segmental mobility required for effective thermally activated shape-memory behavior.

#### 3.4.3. Temperature-Dependent Thermomechanical Response of the Hybrid Networks

The DMA results provide insight into the temperature-dependent thermomechanical response of the PDMS–PCLDMA hybrid networks. The covalent network formed by PDMS-MMA, TEGDMA, TMPTMA, and the methacrylate-connected PCLDMA chains defines the permanent network and provides elastic restoring force [15]. In contrast, the semicrystalline PCLDMA domains function as reversible switching domains responsible for temporary shape fixation. In this hybrid network, the covalently crosslinked framework formed by PDMS-MMA, TEGDMA, TMPTMA, and methacrylate-functionalized PCLDMA chains acts as the permanent network that maintains the original geometry and provides elastic restoring force. The flexible PDMS-MMA segments contribute to network elasticity, while the multifunctional TMPTMA crosslinking points enhance structural integrity. Although PCLDMA chains are chemically incorporated into the permanent network through methacrylate functionalization, their semicrystalline domains provide reversible switching behavior through temperature-dependent melting and recrystallization. During thermal programming, the increased mobility of PCLDMA segments allows deformation, while subsequent cooling enables temporary shape retention through recrystallization of the switching domains. Upon reheating, the elastic energy stored in the permanent network drives recovery toward the original shape. The temperature-dependent decrease in storage modulus indicates progressive thermal softening of the photocrosslinked network, which facilitates deformation and subsequent recovery upon heating. PCLDMA melting and recrystallization may contribute to this thermally activated response; however, their specific contribution cannot be conclusively established without direct calorimetric characterization [33].

The macroscopic recovery observed upon heating is consistent with a thermally activated softening process. However, in the absence of direct calorimetric characterization, the relative contributions of PCLDMA melting/recrystallization and general network softening cannot be quantitatively distinguished in the present study. The temperature-dependent decrease in storage modulus indicates progressive thermal softening of the photocrosslinked network, which facilitates deformation and subsequent recovery upon heating. Overall, the thermomechanical response reflects the combined influence of PDMS-MMA-containing flexible segments, network constraint associated with TMPTMA content, and temperature-dependent softening of the PCLDMA-containing network. PDMS-MMA enhances chain mobility and recovery kinetics, whereas TMPTMA strengthens the permanent network and improves elastic energy storage. Accordingly, the present DMA results are interpreted primarily in terms of temperature-dependent network softening rather than as direct evidence of a specific crystallization-controlled switching mechanism.

### 3.5. Properties of the PDMS–PCLDMA Hybrid Networks

**Figure 7:**
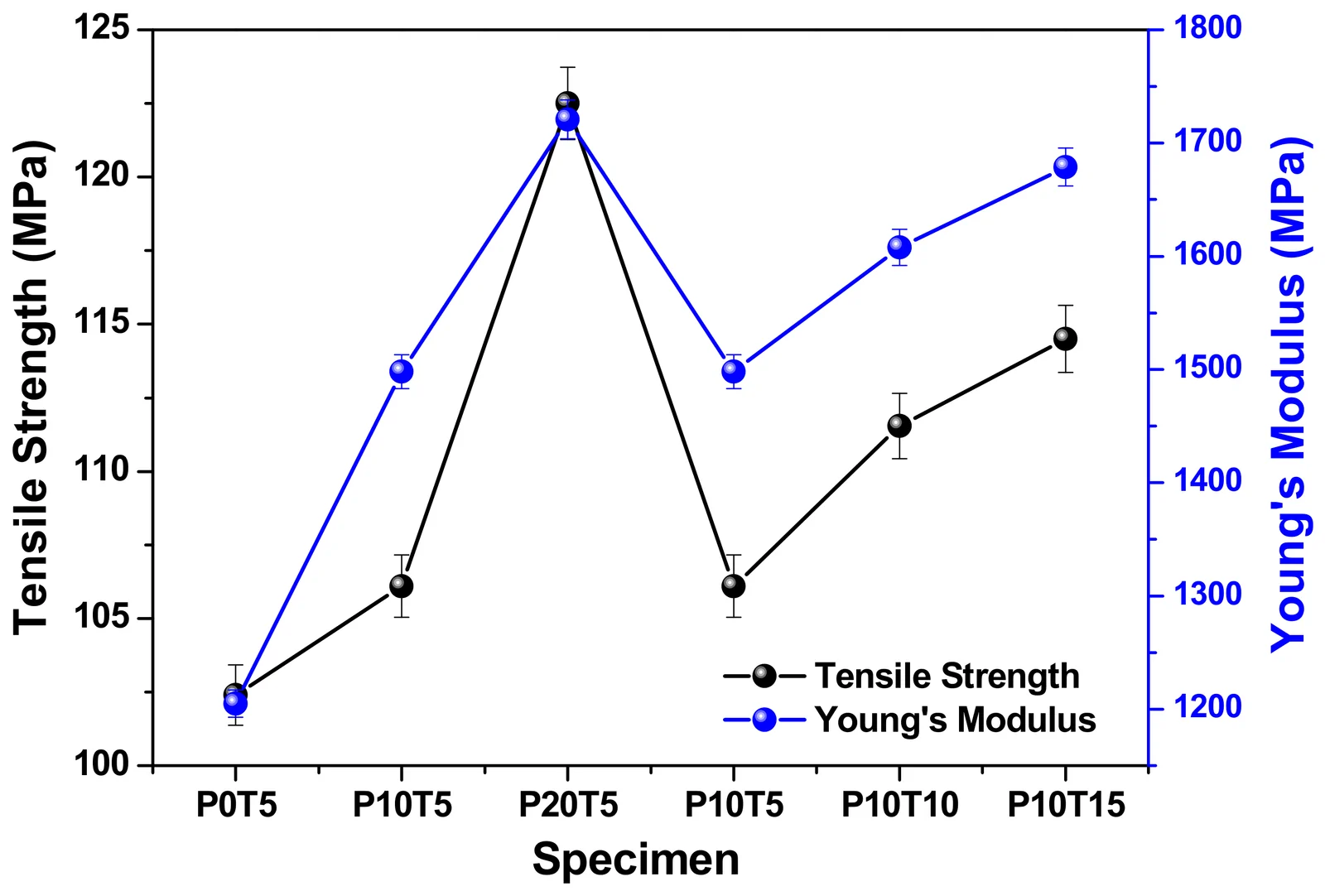
Tensile strength and Young’s modulus of the SLA-printed PDMS–PCLDMA hybrid networks.

The mechanical properties of the SLA-printed PDMS–PCLDMA hybrid networks were evaluated by uniaxial tensile testing. Tensile strength and Young’s modulus are important parameters for shape memory materials because the printed structures must withstand deformation during programming while maintaining sufficient elastic integrity for shape recovery. In particular, the balance between stiffness, strength, and flexibility is critical for thermally responsive 4D-printed structures. As shown in Figure 7 and Figure S1, the tensile properties were strongly affected by both PDMS-MMA content and TMPTMA concentration. Increasing the PDMS-MMA content from P10T5 to P20T5 resulted in a gradual increase in both tensile strength and Young’s modulus. This trend indicates that PDMS-MMA was effectively incorporated into the photocured network and contributed to mechanical reinforcement rather than simply acting as a soft diluent. Although PDMS-MMA contains flexible siloxane segments, the measured increase in Young’s modulus indicates that the macroscopic mechanical response cannot be described by a simple flexibilizing effect of PDMS-MMA alone. The improved mechanical performance of P20T5 can also be associated with the hybrid network structure formed by PDMS-MMA, PCLDMA, TEGDMA, and TMPTMA. The flexible PDMS-MMA segments can help dissipate stress and reduce brittleness, while the covalently crosslinked acrylate/methacrylate network provides structural support. In addition, PCLDMA segments may contribute to physical reinforcement through semicrystalline domains. Therefore, the increase in tensile properties with PDMS-MMA content suggests that the elastomeric and semicrystalline components are effectively combined within the printed network. The increase in TMPTMA content also enhanced the mechanical properties. As the TMPTMA concentration increased, both tensile strength and Young’s modulus increased, indicating that the higher crosslinker content strengthened the covalent network. This result is consistent with the gel fraction and swelling measurements discussed in Section 3.2, where increasing TMPTMA content increased gel fraction and reduced swelling ratio. The denser network formed at higher TMPTMA content can more effectively resist deformation, leading to improved stiffness and tensile strength. However, the role of TMPTMA must be interpreted carefully in the context of shape memory behavior. A higher crosslink density improves mechanical strength and elastic energy storage, which can be beneficial for shape recovery. Nevertheless, excessive crosslinking may restrict the mobility of PCLDMA segments and restrict the segmental mobility associated with temporary-shape fixation. Therefore, the mechanical improvement obtained by increasing TMPTMA should be balanced against the need to preserve sufficient PCLDMA mobility for thermally reversible switching. Overall, the tensile testing results demonstrate that the PDMS–PCLDMA hybrid networks possess sufficient mechanical robustness for shape memory programming and recovery. PDMS-MMA contributes to a flexible yet mechanically stable network, while TMPTMA increases stiffness and strength by enhancing covalent crosslinking. Together with the network, swelling, shrinkage, and DMA results, the tensile properties further support the formation of mechanically robust SLA-printable hybrid networks suitable for thermally triggered shape memory applications. 

### 3.6. Shape Memory Performance and Functional Interplay

**Figure 8:**
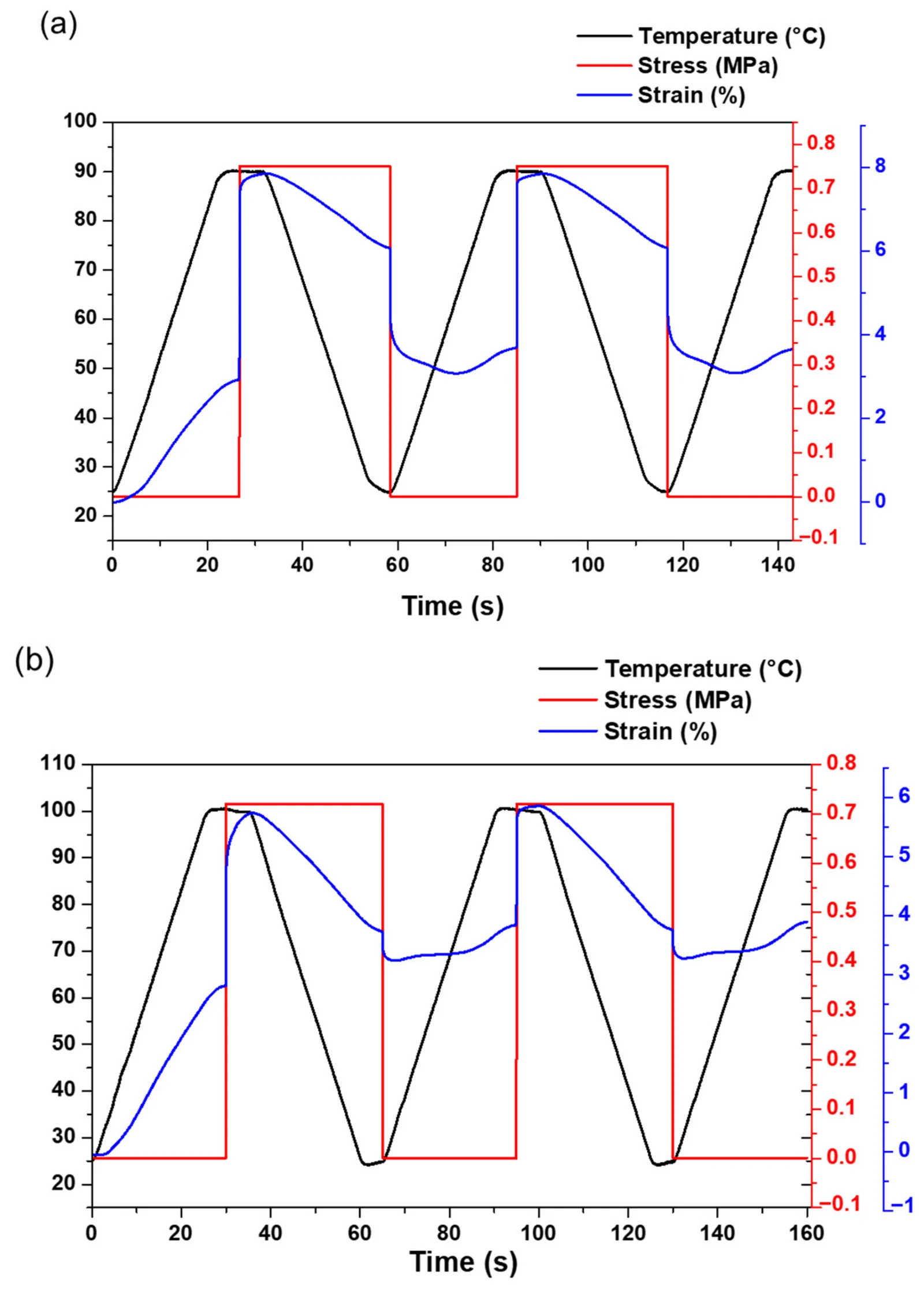
Shape memory properties of representative formulations: (**a**) P20T5 and (**b**) P10T15.

**Figure 9:**
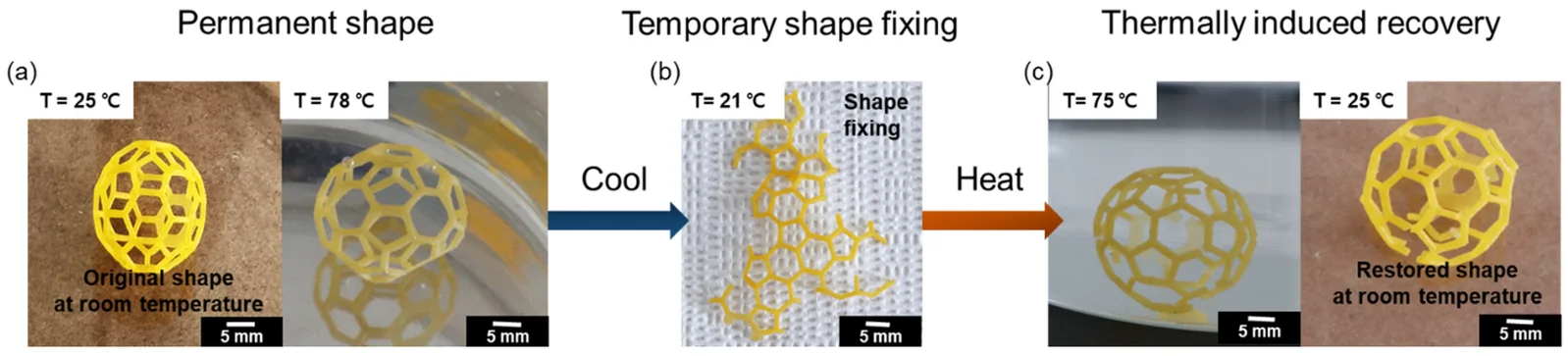
(**a**) Permanent shape of the SLA-printed PDMS–PCLDMA lattice structure at room temperature and thermally assisted deformation after heating to 78 °C, near the PCLDMA switching transition. (**b**) Temporary shape fixation after cooling to 21 °C under constraint. (**c**) Shape recovery upon reheating to 75 °C, followed by retention of the restored shape after cooling back to room temperature. The lattice structure was used to demonstrate the macroscopic thermal shape-recovery capability, whereas quantitative shape-memory parameters were determined from rectangular specimens.

Based on printability, structural integrity, and testing reliability, P20T5 and P10T15 were selected as representative formulations for quantitative one-way shape memory evaluation. P20T5 represents the high-PDMS-MMA formulation with enhanced flexibility and reduced shrinkage, whereas P10T15 represents the high-TMPTMA formulation with increased network compactness and mechanical stiffness. The specimens were programmed at 90–100 °C and cooled to approximately 20 °C under force-controlled loading to fix the temporary shape, followed by unloaded recovery at 90–100 °C. Cyclic thermomechanical tests were performed using rectangular specimens to quantitatively evaluate shape fixity and shape recovery behavior. The calculated shape fixity and recovery ratios are summarized in Table 4, and the corresponding strain evolution during programming and recovery cycles is shown in Figure 8. The P20T5 formulation showed shape fixity values of 95.96% and 99.68% in the first and second cycles, respectively. Similarly, P10T15 maintained high fixity values of 94.02% and 94.85%, demonstrating that even the formulation with higher TMPTMA content retained sufficient temporary-shape stability. The shape recovery behavior showed a more pronounced dependence on network composition. P20T5 exhibited a recovery ratio of 84.10% in the first cycle, which increased to 99.07% in the second cycle. The relatively high recovery performance of P20T5 may be associated with its less constrained network structure, as indicated by its higher swelling ratio and lower network rigidity. The increased molecular mobility provided by PDMS-MMA-containing flexible segments may facilitate thermally induced rearrangement during recovery. Because only two thermomechanical cycles were evaluated and no dedicated training or mechanical-hysteresis analysis was performed, the origin of this cycle-dependent difference cannot be resolved from the present data. The observed increase in second-cycle recovery is therefore reported as an experimental observation rather than as evidence of a training effect or structural relaxation mechanism. These results demonstrate thermally activated one-way shape-memory behavior in the two selected formulations, while the specific molecular origin of the differences between formulations requires further investigation. PDMS-MMA contributes flexible elastic domains that assist recovery, whereas PCLDMA-containing segments may contribute to temporary-shape fixation. TMPTMA improves network strength and permanent-shape stability, but excessive crosslink density can restrict the segmental mobility required for efficient recovery. These quantitative shape-memory parameters were obtained from rectangular specimens and represent the thermomechanical response of the resin under the applied testing conditions, rather than direct measurements of the printed lattice structure shown in Figure 9. Beyond quantitative thermomechanical cycling, the shape-memory capability of the developed resin was further demonstrated using an SLA-printed lattice structure. Unlike the rectangular specimens used for quantitative SME evaluation, the lattice structure was employed for qualitative visualization of macroscopic shape recovery behavior [41]. Overall, the shape memory results support the proposed structure–property relationship. Recent advances in 4D printing have highlighted the importance of programmable polymer networks that combine printability with thermally activated shape transformation for applications such as deployable devices and biomedical systems [14,42]. In this context, the present SLA-printable hybrid resin demonstrates the potential of integrating flexible silicone segments, semicrystalline switching domains, and covalent network constraints to achieve printable and thermally responsive architectures. Among the two formulations evaluated quantitatively, P20T5 exhibited high shape fixity and nearly complete second-cycle recovery. P10T15 also exhibited high fixity and high second-cycle recovery, whereas the lower first-cycle recovery indicates a cycle-dependent response. Similar increases in recovery ratio after the initial cycle have been reported in thermally activated SMP systems [43]. The origin of this difference could not be resolved from the present measurements. The macroscopic one-way shape-memory behavior of the SLA-printed lattice structure was qualitatively demonstrated using the P10T15 formulation, as shown in Figure 9. As shown in Figure 9a, the printed lattice retained its permanent shape at room temperature and became deformable after heating at 78 °C, where the printed network exhibited sufficient thermal softening for deformation. The deformed structure was then cooled to 21 °C under constraint, and the temporary shape was fixed after cooling, as shown in Figure 9b. The temporary geometry remained stable after cooling under constraint. Upon reheating to 75 °C, the fixed lattice recovered toward its original geometry, and the restored shape was maintained after cooling back to 25 °C, as shown in Figure 9c. The lattice structure was fabricated to visualize the macroscopic shape-recovery capability of the developed SLA-printable resin. Unlike the quantitative SME evaluation described above, the lattice structure was evaluated qualitatively to visualize its shape recovery behavior.

## 4. Conclusions

This study demonstrates a formulation-dependent SLA-printable PDMS–PCLDMA hybrid resin platform exhibiting thermally triggered one-way shape-memory behavior. The designed resin system combines flexible siloxane-containing segments with PCLDMA-containing photocurable components and a tunable methacrylate-crosslinked network, enabling composition-dependent control of printability, network formation, dimensional fidelity, thermomechanical response, and shape-memory performance. The network analysis confirmed that all printed formulations formed stable covalent networks with gel fractions above 90%. Increasing PDMS-MMA content enhanced network flexibility and increased solvent uptake, whereas increasing TMPTMA concentration improved gel fraction and reduced swelling by increasing covalent crosslink density. Dimensional analysis further showed that the printed samples maintained low linear shrinkage, with PDMS-MMA incorporation contributing to reduced polymerization shrinkage and improved dimensional fidelity. Thermal and thermomechanical analyses demonstrated that the hybrid networks remained stable within the programming and recovery temperature range. Semicrystalline PCLDMA segments may contribute to the thermally responsive behavior of the network. DMA results further supported the importance of balancing PDMS-MMA-induced mobility with TMPTMA-induced network constraint for achieving effective shape memory actuation. Mechanical testing showed that the hybrid networks possessed sufficient robustness for programming and recovery. Increasing PDMS-MMA content and TMPTMA concentration influenced the stiffness–strength balance of the printed networks, confirming that both soft-segment mobility and crosslink density contribute to the final mechanical response. These mechanical properties are essential for maintaining structural integrity during deformation while allowing recovery upon thermal activation. Among the two formulations evaluated quantitatively, P20T5 exhibited high fixity and nearly complete second-cycle recovery. P10T15 exhibited a lower first-cycle recovery, although the origin of this cycle-dependent difference could not be resolved from the present measurements. These findings provide practical design guidelines for developing 4D-printable silicone–PCLDMA shape-memory materials with tunable network structures and reliable thermally triggered actuation. Overall, this work demonstrates a formulation-based strategy for tuning the network, dimensional, mechanical, and thermally activated shape-memory properties of SLA-printable PDMS–PCLDMA hybrid resins.

## Figures and Tables

**Table 1:** Composition of SLA-printable PDMS–PCLDMA hybrid resin formulations (wt%).

Specimen	PDMS-MMA	PCLDMA (Switching)	TMPTMA (Crosslinker)	Oligomer	TEGDMA (Backbone)	TPO
P0T5	0	30	5	8	55	2
P10T5	10	30	5	8	45	2
P20T5	20	30	5	8	35	2
P10T10	10	30	10	8	40	2
P10T15	10	30	15	8	35	2

**Table 2:** Gel fraction and swelling ratio of SLA-printed PDMS–PCLDMA hybrid networks (*n* = 3).

Specimen	PDMS-MMA (wt%)	TMPTMA (wt%)	Gel Fraction (%)	Swelling Ratio (g/g)
P0T5	0	5	94.2 ± 0.5	1.35 ± 0.04
P10T5	10	5	93.1 ± 0.8	1.48 ± 0.06
P20T5	20	5	91.8 ± 1.1	1.62 ± 0.09
P10T10	10	10	93.5 ± 0.8	1.42 ± 0.04
P10T15	10	15	95.4 ± 0.4	1.32 ± 0.05

**Table 3:** Linear shrinkage of SLA-printed PDMS–PCLDMA hybrid networks (*n* = 3).

Specimen	PDMS-MMA (wt%)	TMPTMA (wt%)	Linear Shrinkage (%)
P0T5	0	5	2.1 ± 0.01
P10T5	10	5	1.9 ± 0.02
P20T5	20	5	1.7 ± 0.01
P10T10	10	10	1.8 ± 0.01
P10T15	10	15	1.8 ± 0.02

**Table 4:** Shape memory properties of representative PDMS–PCLDMA hybrid networks.

Specimen	Shape Fixity, (*R_f_*, %)		Shape Recovery, (*R_r_*, %)	
	Cycle 1	Cycle 2	Cycle 1	Cycle 2
P20T5	95.96	99.68	84.10	99.07
P10T15	94.02	94.85	68.40	97.43

## Data Availability

The original contributions presented in this study are included in the article/Supplementary Material. Further inquiries can be directed to the corresponding author.

## Supplementary Materials

The following supporting information can be downloaded at https://www.mdpi.com/article/10.3390/polym18172095/s1. Figure S1. Tensile stress–strain curves of SLA-printed specimens with different resin formulations. (a) Effect of PDMS-MMA content and (b) effect of TMPTMA content on the tensile deformation behavior.
