# Supplementary material for: Thermally Activated Shape-Memory Behavior of SLA-Printed PDMS–PCLDMA Hybrid Networks
Source: Polymers (Basel). 2026 Aug 28;18(17):2095. doi: 10.3390/polym18172095 (PMC13568445; doi:10.3390/polym18172095)
Supplement: Supplementary file 1 [file polymers-18-02095-s001.zip › polymers-4530501-supplementary.pdf]

polymers

MDPI

Supplementary Materials

Enhancing Mechanical Strength of Photocurable 3D Printing resin by Potassium Titanate Additives

Yura Choi, and Namchul Cho*

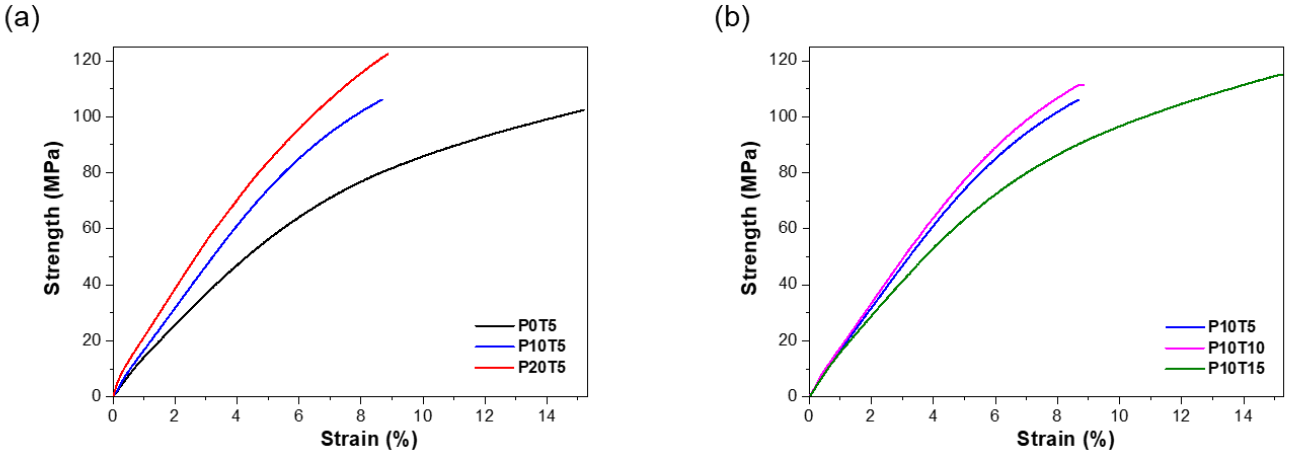

Figure S1. Tensile stress–strain curves of SLA-printed specimens with different resin formulations. (a) Effect of PDMS-MMA content and (b) effect of TMPTMA content on the tensile deformation behavior.
